# Diagnosing Congenital Diaphragmatic Eventration Presenting With Transient Cardiac Dextroposition in an Adult: A Case Report and Literature Review

**DOI:** 10.1155/crpu/1851328

**Published:** 2026-08-05

**Authors:** Yeganeh Rafiei, Behnaz Movahedi, Fatemeh Bayat, Fatemeh Zohrian

**Affiliations:** ^1^ Student Research Committee, School of Medicine, Alborz University of Medical Sciences, Karaj, Iran, abzums.ac.ir; ^2^ Rajaei Clinical Research Development, Alborz University of Medical Sciences, Karaj, Iran, abzums.ac.ir; ^3^ Cardiovascular Research Center, Alborz University of Medical Sciences, Karaj, Iran, abzums.ac.ir

## Abstract

Congenital diaphragmatic eventration (CDE) is a rare developmental anomaly characterized by abnormal elevation of an intact hemidiaphragm due to deficient muscular development. Although often asymptomatic in adults, it may present with respiratory or gastrointestinal complaints. We report a 65‐year‐old man with congenital left hemidiaphragmatic eventration associated with gastric displacement and colonic interposition. He initially presented with chest pain and was found to have cardiac dextroposition. Multimodality imaging, including fluoroscopy, demonstrated a transient rightward cardiac displacement that became more apparent after postprandial gastric distention and regressed as gastric decompression occurred. This dynamic finding suggests that gastrointestinal distention and associated mass effect can exacerbate mediastinal displacement in patients with CDE. This case highlights the importance of considering congenital diaphragmatic anomalies in adults with atypical chest symptoms and emphasizes the value of dynamic imaging in clarifying reversible cardiac displacement.

## 1. Introduction

Congenital diaphragmatic eventration (CDE) is a rare developmental anomaly characterized by the permanent elevation of an anatomically intact, yet functionally deficient, hemidiaphragm. Unlike congenital diaphragmatic hernia, in which a discrete defect exists, CDE involves a large, thin, fibroelastic portion of the diaphragm that fails to undergo normal muscular development [1, 2]. While CDE is often discovered incidentally in adulthood, it can produce a spectrum of respiratory, gastrointestinal, or even cardiac symptoms, potentially mimicking more severe thoracic pathology.

Diagnostic differentiation between CDE, diaphragmatic paralysis, and primary pulmonary anomalies, such as lobar agenesis, is crucial for appropriate clinical management. As our understanding of CDE has evolved, it has become evident that this condition may be associated with other congenital findings, including megacolon and gastric malposition, further complicating its clinical presentation [3, 4]. However, the literature regarding the hemodynamic implications of diaphragmatic anomalies—specifically regarding transient mediastinal shifts—remains sparse.

In this report, we present a rare case of a 65‐year‐old male initially suspected of having pulmonary lobar agenesis due to persistent cardiac dextroposition. Through a comprehensive, dynamic diagnostic work‐up—including fluoroscopy, MRI, and computed tomography (CT)—we identified an underlying CDE as the etiology. Notably, our findings reveal a reversible, postprandial cardiac dextroposition induced by gastrointestinal distention, a pathophysiological mechanism that provides novel insight into the clinical presentation of CDE in adult life.

## 2. Case Presentation

A 65‐year‐old male smoker presented to the Emergency Room with chest pain. The pain was a constant retrosternal pain with radiation to the left arm and jaw. He had a past medical history of myocardial infarction (MI) 3 years ago but denied any history of recurrent respiratory infections or COVID‐19. There is no trauma history (car accidents, motor accidents, falling downs, etc.) Physical examination revealed mildly asymmetrical chest movement and absent breath sounds on the right side, but the heart sounds were heard on the middle to the left side of the chest. Initial work‐up illustrated an NSTEMI on ECG and echocardiography illustrated heart displacement and mild left ventricular systolic dysfunction (LVEF = 45*%*). An angiography revealed three‐vessel disease, and coronary artery bypass grafting (CABG) was planned.

**Figure 1:**
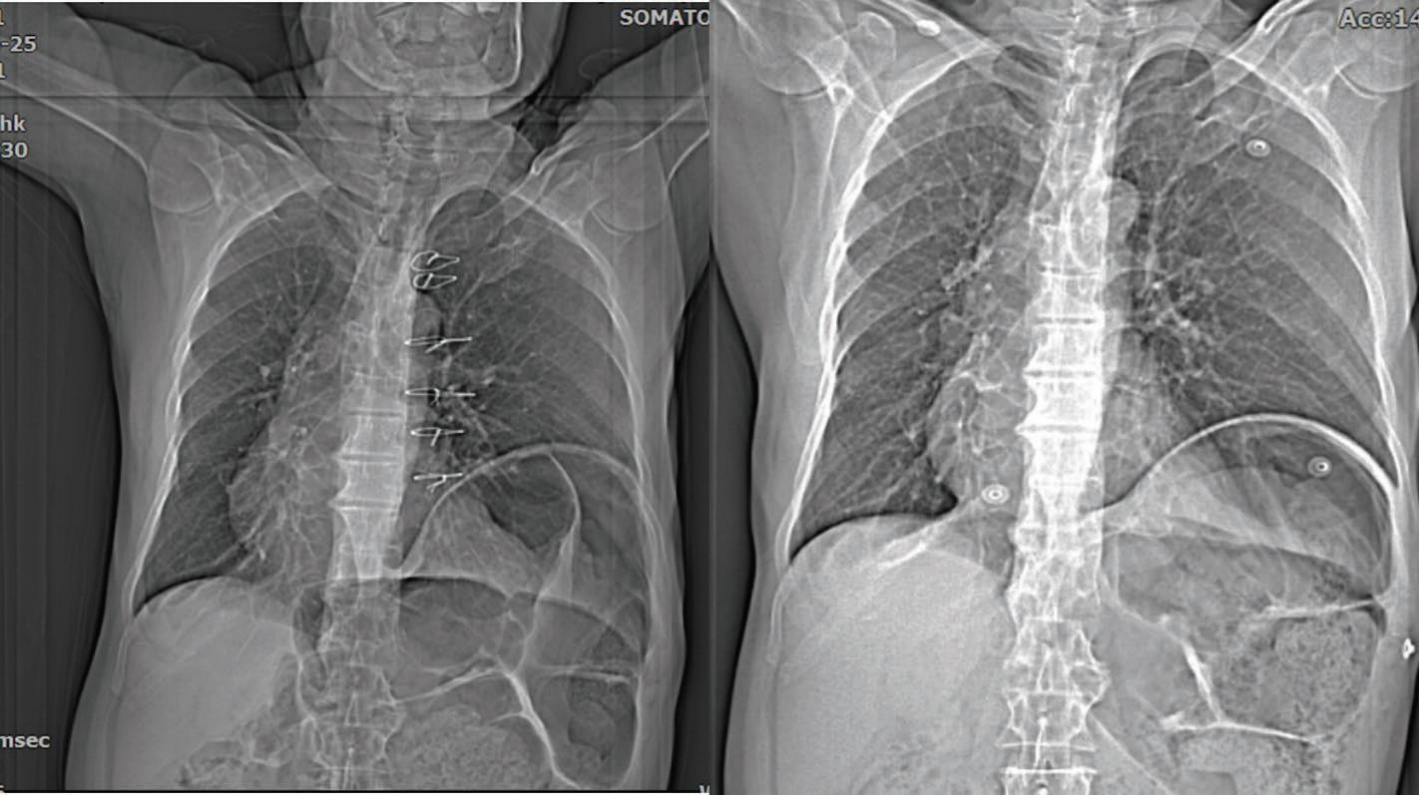
Chest CT scan (coronal view). Dynamic heart position with post‐prandial, 2 years post operation (left) and pre‐prandial, pre operation (right).

**Figure 2:**
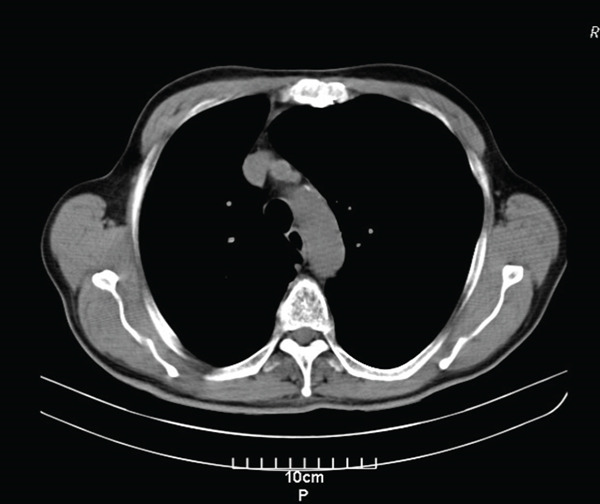
Chest CT scan, mediastinal view, hyperinflation of the left lung and the normal position of the aorta that rules out dextrocardia.

**Figure 3:**
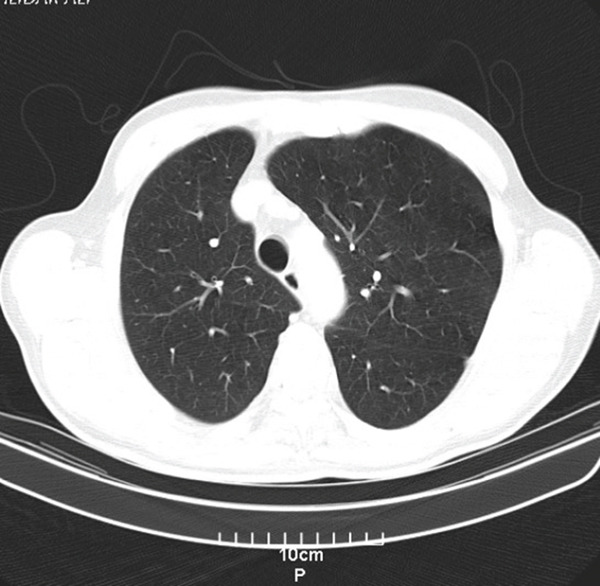
Chest computed topography (CT scan), hyperinflation of the left lung, and shifting the mediastinum to the right.

**Figure 4:**
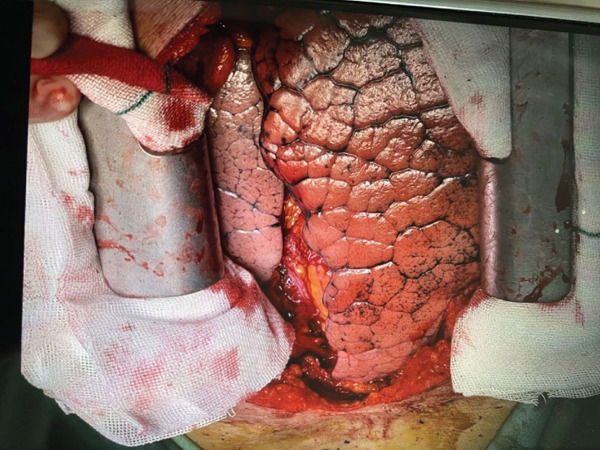
Hyperinflation of the left lung is seen during CABG surgery.

**Figure 5:**
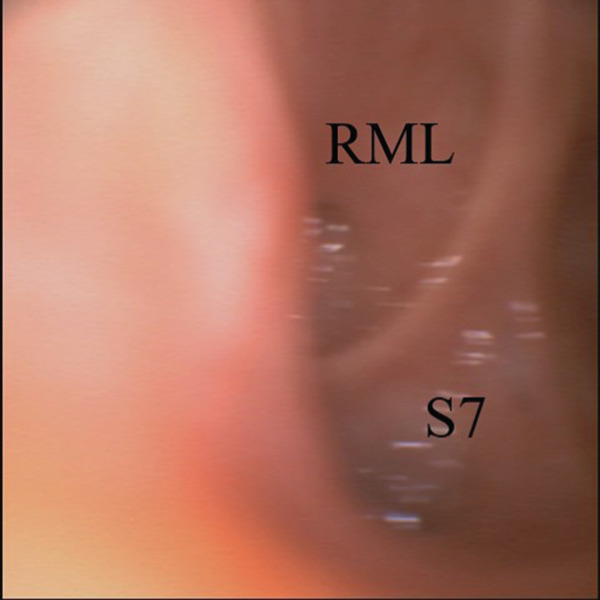
Bronchoscopy view, indicating the absence of right main bronchus.

Because of an unforeseen technical failure of the radiography unit at the time of initial presentation, a chest radiograph was not available. Given the clinical suspicion of significant intrathoracic pathology and the need for urgent evaluation of the patient’s chest pain, we proceeded directly to contrast‐enhanced CT of the chest and abdomen for definitive diagnostic characterization. Preoperative CT scans showed an elevated left hemidiaphragm, but the initial interpretation did not fully describe the diaphragmatic anomaly (Figures 1 and 2). The surgeon, during the CABG procedure, noted severe dextroposition of the heart and hyperinflation of the left lung (Figures 3 and 4). This was initially attributed to hypoplasia or agenesis of the right middle lobe of the lung, a diagnosis that was supposedly reinforced by an immediate post‐operative bronchoscopy revealing atresia of the RMB and the 7th segment (S7) (Figure 5). The patient also underwent pulmonary function tests (PFTs), which revealed a moderately obstructive pattern.

**Figure 6:**
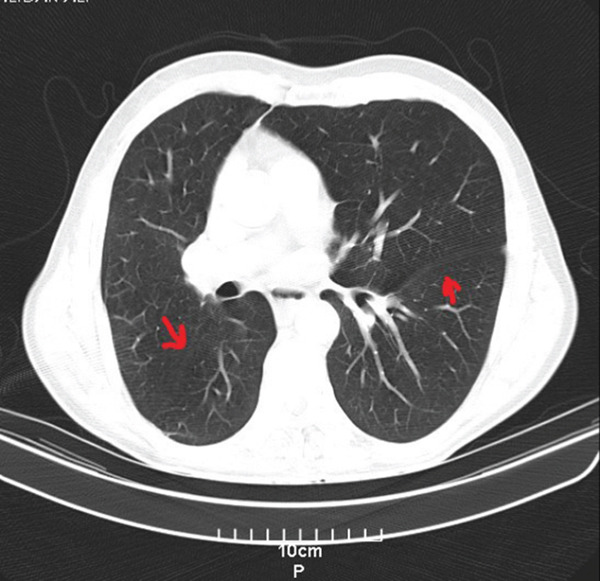
Chest CT scan, dispositioning of the right fissure.

Two years later, the patient returned for follow‐up and consulted with new symptoms of post‐prandial heartburn. In addition, more extensive diagnostic work‐up was performed to investigate these new symptoms. This new imaging, including the coronal chest CT scan, showed that the right middle lobe was not absent but rather had an anomalous posterior location (Figure 1). The medial and lateral segments of the right middle lobe were in their normal relationship to each other but the whole lobe was in the posterior position, i.e., it was not interposed between the upper and lower lobes (Figure 6). Imaging demonstrated congenital eventration of the left hemidiaphragm with upward displacement of the stomach and adjacent colonic segments into the subdiaphragmatic space beneath the elevated hemidiaphragm.

**Figure 7:**
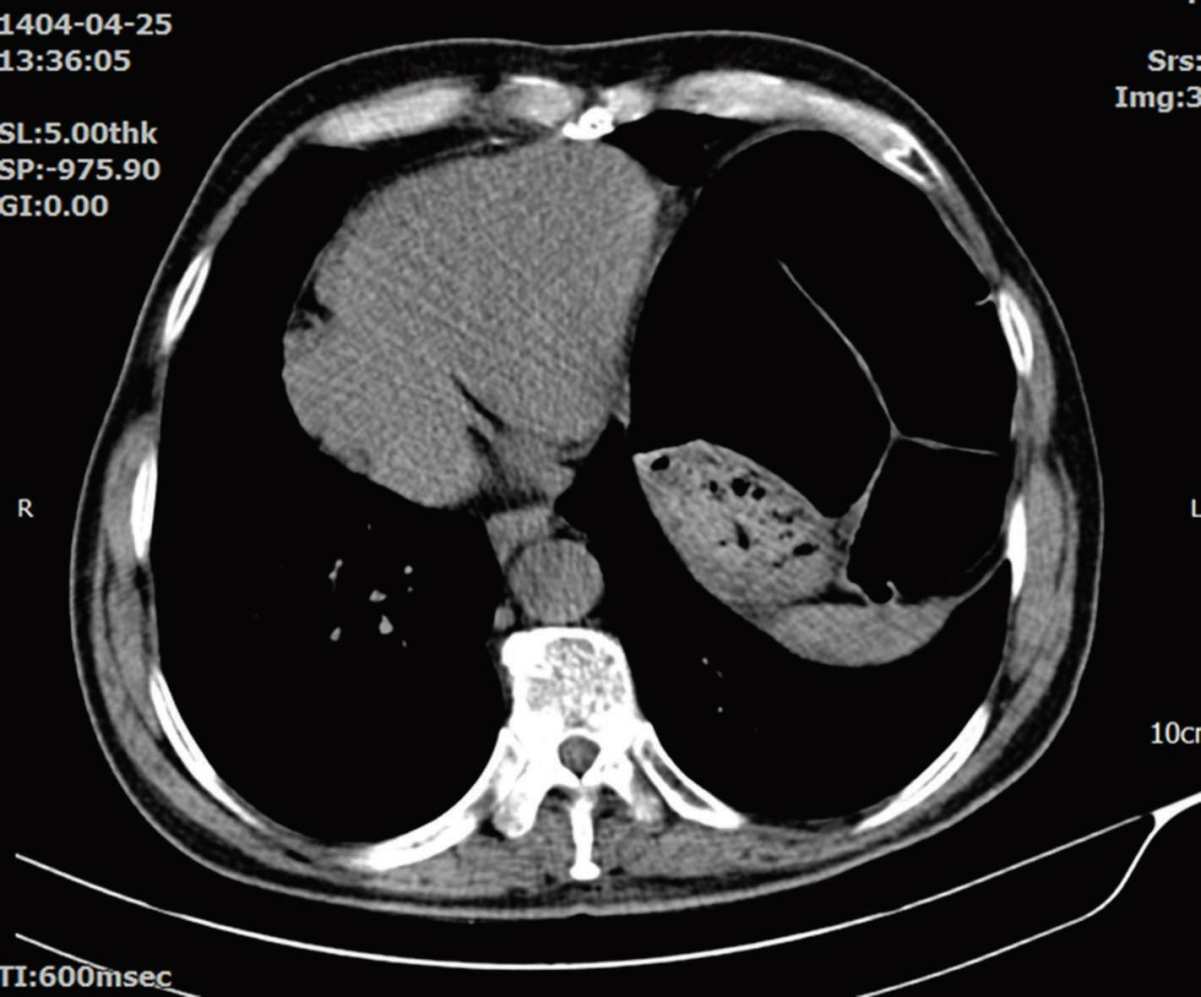
Chest CT scan (axial view). Congenital diaphragmatic eventration with intrathoracic herniation of dilated colon (megacolon) in the left hemithorax causing mediastinal shift.

**Figure 8:**
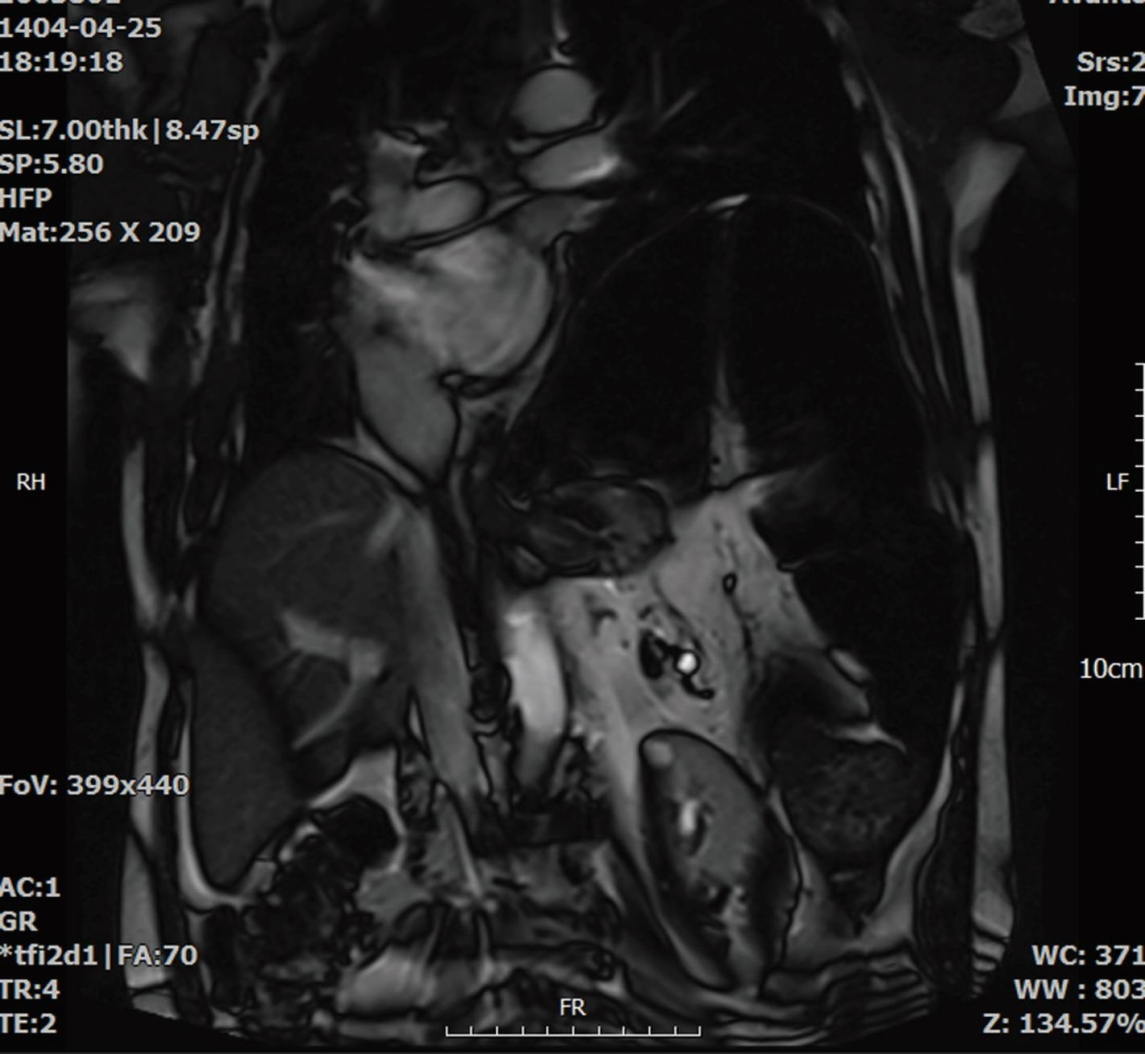
Coronal MRI scan. Demonstrating congenital diaphragmatic eventration with herniation of dilated bowel loops into the thoracic cavity.

To assess the new post‐prandial symptoms and to exclude the diagnosis, an ultrasound, a fluoroscopy and MRI with dynamic imaging were performed. These tests revealed that when the stomach distended following meals, the intra‐abdominal pressure was increased and pushed the colon upwards, causing the heart to shift temporarily to the right (dextroposition) (Figures 7 and 8). When the gastrointestinal tract contents emptied, the heart returned to its original position (Figure 1). This meal‐induced, dynamic cardiac displacement was an uncommon and unusual feature that provided a novel, central explanation for the patient’s cardiac displacement that was earlier noted intraoperatively. They also ruled out one more differential diagnosis, which was diaphragmatic paralysis.

Abdominal CT scan also performed to rule out the other probable causes of colonic distention. It demonstrated chronic colonic dilation without transition point or signs of obstruction. Electrolyte, thyroid, and inflammatory markers were normal. Finally, the absence of obstructive pathology and lack of acute symptoms, the megacolon was assessed as a chronic incidental finding rather than the cause of the patient’s presentation.

After finalizing the evaluation, we prescribed a proton‐pump inhibitor (PPI) for management of the possible gastrointestinal reflux and a laxative to improve bowel movements and prevent constipation. Smoking cessation and avoiding strenuous resistance exercise were also recommended.

## 3. Discussion

The initial diagnosis of pulmonary lobar agenesis had been based on bronchoscopy findings and the assumption that the dextroposition of the heart was a fixed congenital anomaly. The additional, more comprehensive imaging and reconsideration of the patient’s history, however, identified a different, and even rarer, congenital anomaly: CDE.

CDE is an uncommon developmental anomaly characterized by an abnormal elevation of an intact hemidiaphragm due to partial or complete replacement of muscular fibers by thin fibroelastic tissue that is typically asymptomatic and discovered incidentally in adulthood [1, 2, 5, 6]. Unlike diaphragmatic hernia, the continuity of the diaphragm is preserved, although its structural integrity and contractile function are compromised [5, 6]. Differential diagnoses include diaphragmatic paralysis, subphrenic pathology, pulmonary hypoplasia, and congenital lung anomalies [4, 7]. In particular, diaphragmatic paralysis may mimic eventration on static imaging; however, dynamic evaluation typically demonstrates paradoxical diaphragmatic motion during inspiration [8]. The diagnostic importance of dynamic fluoroscopy and ultrasonography in distinguishing diaphragmatic eventration from paralysis has also been emphasized in recent literature [9]. Another relevant aspect of this case is the coexistence of gastrointestinal involvement, including displacement of abdominal viscera beneath the elevated diaphragm. Previous reports have described associations between diaphragmatic eventration and gastrointestinal abnormalities such as gastric volvulus or megacolon [3, 4]. The occurrence of diaphragmatic eventration with megacolon in our patient is therefore consistent with other observations. The finding of an abnormally located right middle lobe is also a new and interesting finding, further supporting the presence of multiple congenital developmental anomalies.

The most striking and, to our knowledge, unprecedented finding in this case is the transient nature of the cardiac dextroposition. To the best of our knowledge, the literature contains reports of fixed cardiac dextroposition or pseudo‐dextrocardia associated with diaphragmatic elevation [10–12], but reports describing a clearly reversible, postprandial cardiac dextroposition in adult CDE are exceedingly limited. The physiological respiratory motion leads to mild displacement of abdominal organs, true cardiac dextroposition requires a more substantial shift. In our patient, the dynamic fluoroscopy showed a rightward displacement for exceeding normal respiratory variation, supporting a pathological rather than physiological process. This pathophysiology is new and explains the patient’s initial complaint of chest pain and provides a direct link between the gastrointestinal tract and the cardiac displacement. It highlights how the pressure exerted by the distended stomach and colon can transiently compress the heart, leading to a temporary dextroposition.

Adult management recommendations support surgical plication in patients with persistent dyspnea, recurrent pulmonary infections, or progressive mediastinal shift [13]. Our patient did not demonstrate these indications, supporting a conservative strategy.

This case illustrates the importance of a diligent and stepwise diagnostic process, especially in patients with atypical clinical presentations. While the findings on presentation were indicative of a diagnosis of lung agenesis, the recent symptoms and the subsequent, more sophisticated imaging studies developed a more refined and deeper insight into the patient’s anomaly. Sophisticated imaging techniques like fluoroscopy, MRI, and sonography were helpful in illustrating the dynamic nature of the anomaly [7, 14].

A review of the case report literature of CDE in adults and its anomalies reveals a variety of presentations. The majority of reported cases is left‐sided and typically involves the stomach or colon with heterogeneous presentations. A summary of relevant cases is presented in Table 1.

**Table 1:** Adult congenital diaphragmatic eventration case reports (English‐language; PubMed/PMC/Scopus) through Aug 16, 2025 [14, 15].

Author (year)	Country	Age/sex	Side	Presentation	Diagnostic highlights	Management	Outcome/follow‐up	Dextropositioning of the heart	Notes
Mantoo and Mak (2007) [14]	Singapore	51 M	Left	Dyspnea; diagnostic dilemma	CXR + CT; intact hemidiaphragm; no trauma	Conservative	Improved with supportive care; plan for plication	No	Diagnostic pitfalls highlighted
Srikrishna et al. (2010) [16]	India	~70s F	NR	Breathlessness	Imaging consistent with congenital eventration	Surgical repair (plication)	Symptom relief	No	“Septuagenarian lady”
Shen and Che (2012) [17]	China	35 M	Left	Progressive dyspnea	Imaging; congenital DE confirmed intra‐op	Plication	Clinical improvement	No	Clear adult congenital DE
Visouli et al. (2012) [18]	Greece	Adult	Left	Symptomatic dyspnea	Imaging + intra‐op confirmation	VATS plication	Symptom and radiographic improvement	No	Technique‐focused
Guzman et al. (2017) [19]	Philippines	32 F	Left	Dyspnea, GI symptoms	CXR, CT, barium swallow	Abdominal plication	Symptom‐free at 2 years	No	Classic imaging series
Makwana et al. (2017) [20]	India	58 F	Right (complete)	Dyspnea	CXR/CT	Surgical plication planned	Stable, favorable outcome	No	Rare complete right eventration
Forter‐Chee‐A‐Tow and Smith (2023) [21]	Barbados	79 M	Left	Dyspnea	Imaging excluding hernia	Thoracoscopic plication	2‐year follow‐up, improvement	No	Durable outcome
Xu and Yu (2024) [15]	China	74 F	Left (localized→complete)	Post‐COVID dyspnea	Serial CXR/CT: progression	Thoracic plication	Good recovery at 6 months	No	First COVID‐associated progression
Gupta et al. (2023) [22]	India	45 M	Left	Abdominal pain, bloating	CT; eventration with GI symptoms	Laparoscopic plication	Symptom‐free at 1 year	No	GI symptoms prominent
Mir et al. (2014) [11]	India	70 M	Left	Progressive breathlessness, Type 2 respiratory failure	CXR, CT: complete eventration with herniation of colon and stomach; dextrocardia	Surgical repair (plication)	Improvement with supportive care; plan for plication	Fixed dextrocardia	Complex case with multiple associated anomalies

## 4. Conclusion

Based on our research, this case highlights the extreme rarity of a CDE presenting with a dynamic, post‐prandial cardiac dextroposition in an adult. The initial diagnosis of pulmonary agenesis was superseded by a more comprehensive understanding of the patient’s condition. This case demonstrates the value of advanced and dynamic imaging techniques in elucidating the underlying pathology of complex and atypical presentations. The transient nature of the heart displacement is, to our knowledge, a first‐time report and provides a unique insight into the pathophysiology of diaphragmatic anomalies.

## Funding

No funding was received for this manuscript.

## Consent

The authors have nothing to report.

## Conflicts of Interest

The authors declare no conflicts of interest.

## Data Availability

Research data are not shared.
